# Enhanced coagulation index: a potent prognostic indicator for clinical outcomes in non-metastatic breast cancer following surgery and adjuvant therapy

**DOI:** 10.3389/fonc.2025.1515898

**Published:** 2025-05-29

**Authors:** Bolin Lu, Ji Wu, Mu Yuan, Xuxu Zhang, Xing Qiu, Jianyin Bai, Ming Yao, Sainan You, Shanshan Wang, Linlin Zhen

**Affiliations:** ^1^ The Affiliated Suqian Hospital of Xuzhou Medical University, Suqian, China; ^2^ The Huaian Clinical College of Xuzhou Medical University, Huaian, China

**Keywords:** breast cancer, APTT/TT index, disease-free survival, treatment, prognosis

## Abstract

**Background:**

A significant number of studies have demonstrated a correlation between the prognosis of patients with malignant tumors and a single coagulation marker. However, relatively few studies have examined the correlation between complex coagulation markers. The purpose of this study was to investigate the relationship between the level of complex coagulation markers and nonmetastatic breast cancer patients’ clinical outcomes after receiving comprehensive treatment.

**Methods:**

This retrospective cohort study analyzed the information breast cancer patients treated between January 2016 and December 2018 at the affiliated Suqian hospital of Xuzhou Medical University. Subject-working characteristic curves were used to determine the area under the curve. Multivariate Cox regression models were used to adjust for potential confounders and to assess independent associations between biochemical markers and survival outcomes.

**Results:**

Of the 264 patients with a median age of 48 years, 33 (12.5%) patients experienced a survival event. The X-TILE analysis shows that the best cut-off value for APTT/TT is 1.4, and the 5-year Disease-free survival (DFS) for high APTT/TT (≥1.4) is more limited. The receiver operating characteristic curve decided the APTT/TT performance (AUC=0.685, 95%CI 0.602-0.768). Multivariate Cox regression models showed that increased APTT/TT (HR=4.057, p=0.032) and more lymph node metastases (HR=2.324, p<0.001) were independent prognostic factors for DFS.

**Conclusions:**

This study indicated a pivotal role of the APTT/TT ratio in forecasting the prognosis of breast cancer patients following comprehensive treatment. The findings underscore the utility of integrating coagulation markers, alongside traditional clinicopathological indices, to enhance prognostic assessments in clinical practice.

## Introduction

Breast cancer is the most common cancer and the leading cause of cancer mortality among women worldwide (1). Nonmetastatic breast cancer is treated with postoperative systemic therapies that include chemotherapy, endocrine therapies, immunotherapy with monoclonal antibodies directed at tumor receptors, and radiation. Chemotherapy is the cornerstone of breast cancer treatment. Extensive treatment in view of chemotherapy can be given to reduce the risk of recurrence and distant metastasis (2). Patients’ prognoses can be somewhat predicted by evaluating the benefits of comprehensive treatment. Nonetheless, it isn’t not difficult to evaluate the prognosis of therapies, and the ongoing normally utilized assessment strategies have specific restrictions and lacks, like clinical symptoms, physical examination, imaging examination, pathological examination, tumor marker examination, molecular detection, etc.

Traditional clinicopathological factors like lymph node metastases (LNMs), tumor size, and grade have provided individual prognostic information. Combining multigene gene tests, biomarkers like the estrogen receptor (ER), progesterone receptor (PR), and human epidermal growth factor receptor-2 (HER2), and conventional pathological clinical prognostic factors can result in improved prognostic models (3, 4). Oncotype DX and the Breast Cancer Index are just a few of the gene expression assays that have been shown to accurately define prognosis and have been recommended for clinical use in recent decades (5, 6). However, the costs of these tests are quite high in numerous countries (5, 6). As a result, it is increasingly important to investigate biomarker assays that are straightforward, affordable, and accurate for prognostic purposes.

Numerous studies have demonstrated a close connection between cancer progression and abnormal coagulation function (4, 7). Coagulation-related mechanisms in the tumor stroma and microenvironment can be triggered when growths are available. Tumor patients experience hypercoagulability as a result of the intricate interaction between their malignant tumors and the coagulation system. Furthermore, changing degrees of coagulation status might uncover the basic biological characteristics of cancers. Accordingly, potential indicators for tumor risk stratification include peripheral blood coagulation parameters, which reflect the foundational coagulation state. There is a strong correlation between the clinicopathological characteristics of breast cancer and the levels of thrombin Time (TT) or initiated partial thromboplastin time (APTT) (8). Notwithstanding, current research has largely ignored the combination of different coagulation markers. Moreover, hardly any studies have shown whether coagulation function indicators can direct the prognosis of breast cancer patients after first-line treatment. Subsequently, the connection between prognostic risk of breast cancer and coagulation markers after first-line therapy remains ineffectively comprehended.

This study aimed to assess the prognostic significance of various hematologic markers in predicting DFS in breast cancer patients. By identifying key hematologic indicators, determining optimal cutoff values for screening, and analyzing marker levels following radical surgery combined with adjuvant treatment, the study seeks to enhance the ability to predict patient outcomes.

## Methods

### Study population

We conducted a retrospective analysis of clinical and biochemical data from breast cancer patients treated at Suqian Hospital, affiliated with Xuzhou Medical University, from January 2016 to December 2018. Inclusion Criteria: Female patients aged 18 to 70 years with a diagnosis of stage pT1-4N0-3M0 invasive breast cancer were included in this study. The age range was chosen to encompass a wide demographic of pre-menopausal and post-menopausal women, allowing for a comprehensive evaluation of biochemical markers across varying physiological stages. All patients had a confirmed histological diagnosis of invasive breast cancer through biopsy or surgical specimen analysis. Participants had not undergone any prior systemic or local cancer treatment. Furthermore, they maintained an Eastern Cooperative Oncology Group (ECOG) Performance Status between 0 and 2, indicating acceptable physical condition for treatment. Adequate bone marrow function was confirmed by minimum thresholds for neutrophil count, white blood cell count, platelet count, and hemoglobin level. Additionally, participants demonstrated normal organ function, defined by standard limits for aspartate aminotransferase (AST), alanine aminotransferase (ALT), creatinine, and total bilirubin, with no significant organ impairment. Exclusion Criteria: Patients were excluded from the study if they showed intolerance to chemotherapy or surgical procedures. Additional exclusion criteria included uncontrolled cardiovascular diseases, severe infections, or thrombotic disorders; the presence of inflammatory or metastatic breast cancer at diagnosis; any concurrent malignancy; and known allergies to medications used in the study.

Ethical approval was granted by the affiliated Suqian Hospital of Xuzhou Medical University, and all patients in our study provided informed consent. This study was conducted in accordance with the Declaration of Helsinki.

### Treatment protocol and follow-up

The patient had a major surgical resection and was able to recover quickly. Adjuvant radiotherapy was performed according to the patient’s condition. Patients were treated with one of the following chemotherapy protocols: Cyclophosphamide 600 mg/m² and docetaxel 75 mg/m² IV every 21 days for four cycles; Cyclophosphamide 600 mg/m² and epirubicin 90 mg/m² IV every 21 days for four cycles, followed by either docetaxel 90 mg/m² or paclitaxel 175 mg/m² for four additional cycles, totaling eight cycles.

Patients with HER-2 positive tumors received trastuzumab starting with an 8 mg/kg dose, followed by a maintenance dose of 6 mg/kg every three weeks for one year. Hormone receptor-positive patients underwent endocrine therapy post-chemotherapy, with postmenopausal women receiving an aromatase inhibitor (letrozole 2.5 mg or exemestane 1 mg) daily, and premenopausal women receiving tamoxifen or ovarian function suppression (leuprorelin 3.75 mg or goserelin 3.6 mg every four weeks) in combination with an aromatase inhibitor. DFS was calculated was presented as the time from random a diagnosed until the first recurrence or death.

### Data collection

Baseline data were retrospectively collected at enrollment, including demographic information, tumor size, T stage, N stage, and hormone receptor status (ER, PR, HER2, Ki-67). Additional biochemical markers, including TT, APTT, fibrinogen (FIB), total cholesterol (CHO), triglycerides (TG), high-density lipoprotein (HDL), low-density lipoprotein (LDL), albumin (ALB), alkaline phosphatase (ALP), hematocrit (HCT), lactate dehydrogenase (LDH), and mean corpuscular volume (MCV) were recorded pre-chemotherapy and at various post-chemotherapy intervals. HER-2 status was defined as immunohistochemical3+ or fluorescence *in situ* hybridization amplification per the 2013 American Society of Clinical Oncology (ASCO)/CAP guidelines.

### Statistical analysis

X-tile analysis software version 3.6.1 (Yale University), and IBM SPSS (version 21.0; SPSS Inc, Chicago, IL, USA) were used to analyze data. The continuous parameters were presented as median (interquartile range [IQR]) and were compared using the two-sided Mann–Whitney *U*-test, as well as categorical parameters using the two-sided Chi square test or Fisher’s exact test in two independent samples. X-tile analysis was conducted to identify 5 years DFS as the optimal cut-off values for these factors. Statistical analyses included generating Receiver Operating Characteristic (ROC) curves to determine the area under the curve (AUC) for the biochemical markers. DFS were calculated using the Kaplan–Meier method and were compared between groups using the log rank test. A multivariable Cox regression model was used to adjust for potential confounders and assess the independent association between biochemical markers and survival outcomes. A p < 0.05 was statistically significant.

## Results

### Patients baseline information

264 women with advanced breast cancer were finial included in the study, ranging in age from 24 to 68, with a median age of 48 (IQR 56-67). A survival event occurred in 33 patients (12.5%) over a median follow-up period of 50 months. The low APTT/TT group and the high APTT/TT group did not differ in the clinicopathological parameters of tumor-related factors, such as tumor size, lymph node metastasis, histological grade, HER-2 status, Ki-67 expression, estrogen (ER) and progesterone (PR) receptor status. The median levels of APTT/TT, PLT, and FIB were 1.3 (IQR 1.08-1.49), 230 (IQR 197-273), and 2.45 (IQR 2.19-2.84), respectively. Patients’ characteristics are summarized in **Table 1**.

**Table 1:** Patients characteristics.

Variables	Total (n=264, %)	Low APTT/TT (n=169, %)	High APTT/TT (n=95, %)	*p*
Age, years (median IQR)	48 (43-55)	50 (44-55)	45 (37-51)	0.548^b^
Grade				0.167^a^
G1	4 (1.5)	3 (1.8)	1 (1.1)	
G2	195 (73.9)	129 (76.3)	66 (69.4)	
G3	65 (24.6)	37 (21.9)	28 (29.5)	
T stage				0.788^a^
T1	138 (52.3)	86 (50.9)	52 (54.7)	
T2	112 (42.4)	74 (43.8)	38 (40.0)	
T3	7 (2.7)	4 (2.4)	3 (3.2)	
T4	7 (2.6)	5 (2.9)	2 (2.1)	
N stage				0.648^a^
N0	132 (50.0)	90 (53.2)	42 (44.2)	
N1	83 (31.4)	48 (28.5)	35 (36.8)	
N2	27 (10.3)	20 (11.8)	7 (7.4)	
N3	22 (8.3)	11 (6.5)	11 (11.6)	
ER (+) ^c^	188 (71.2)	123 (72.8)	65 (68.4)	0.453^b^
PR (+) ^c^	172 (65.2)	113 (66.9)	59 (62.1)	0.436^b^
HER-2 (+) ^c^	86 (32.6)	55 (32.6)	31 (32.6)	0.988^b^
Ki-67				0.714^a^
<14%	57 (21.6)	41 (24.3)	16 (16.8)	
14-30%	153 (58.0)	93 (55.0)	60 (63.2)	
≥31%	54 (20.4)	35 (20.7)	19 (20.0)	
APTT/TT (median IQR)	1.30 (1.08-1.49)	1.13 (1.03-1.28)	1.56 (1.47-1.68)	0.018^b^
PLT (median IQR)	230 (197-273)	233 (198-283)	226 (192-260)	0.695^b^
FIB (median IQR)	2.45 (2.19-2.84)	2.44(2.19-2.81)	2.47 (2.19-2.87)	0.167^b^

a Comparison of data between the high and low APTT/TT groups using the two-side Chi square test or Fisher’s exact test.

b Comparison of data between the high and low APTT/TT groups using the Mann-Whitney U test.

c Number of cases were available.

### X-tile analysis to determine the optimal cut-off values

**Figure 1:**
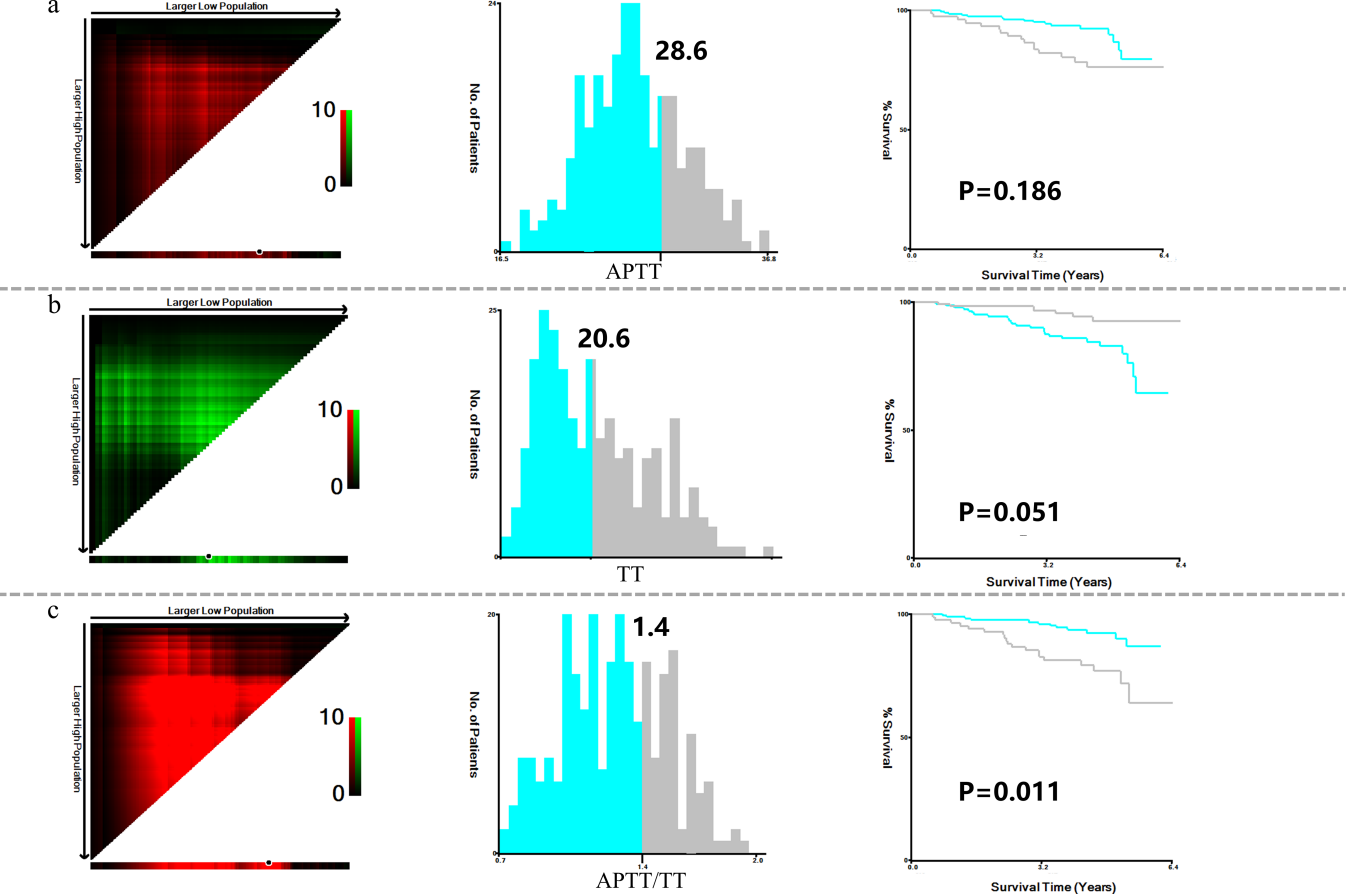
X-title analysis to determine the optimal cut-off values of APTT **(a)**, TT **(b)**, and APTT/TT **(c)**.

**Figure 2:**
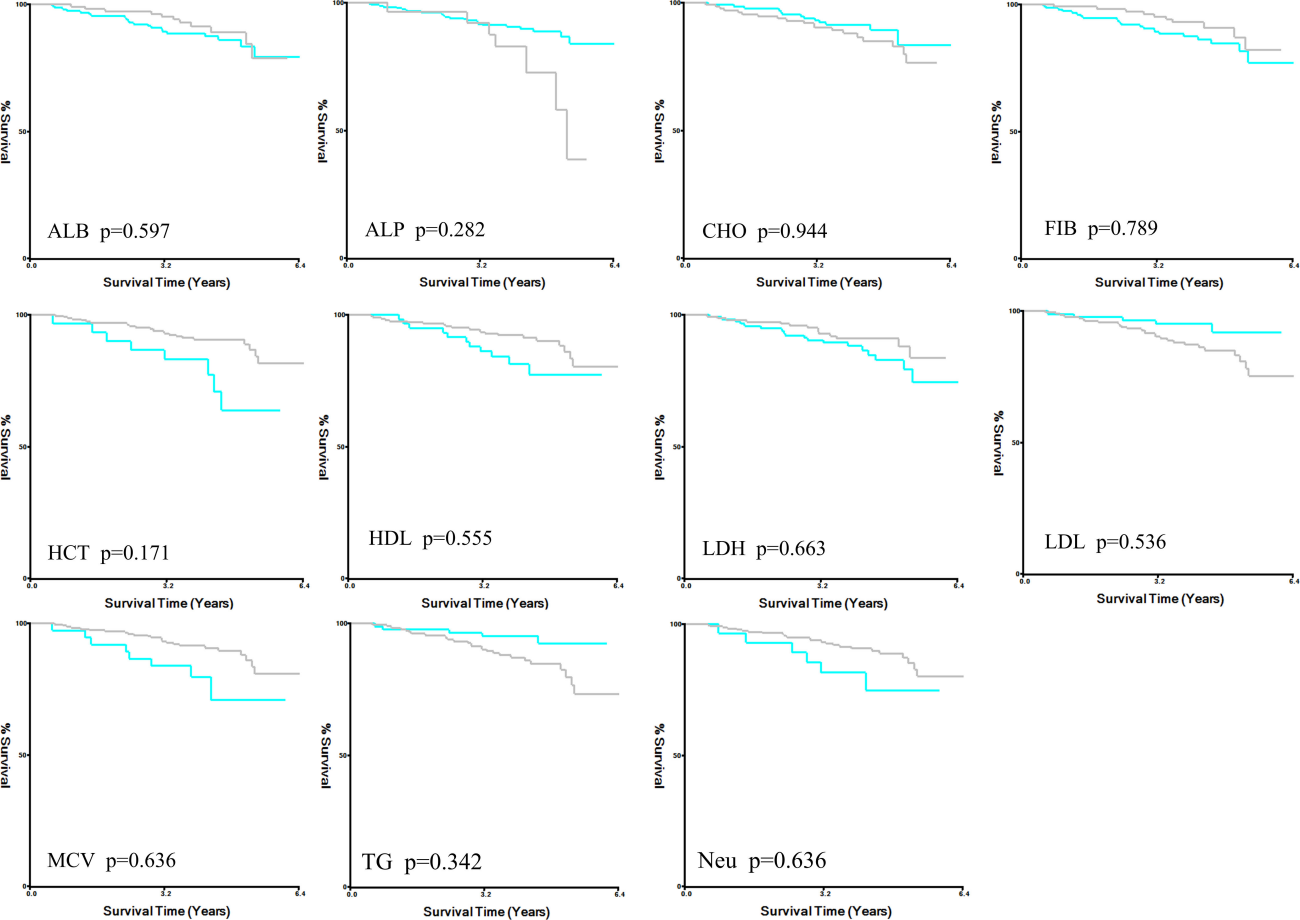
X-title analysis for DFS in ALB, ALP, CHO, FIB, HCT, HDL, LDH, LDL, MCV, TG, and Neu. DFS disease-free survival, ALB albumin, ALP alkaline phosphatase, CHO cholesterol, FIB fibrinogen, HCT hematocrit, HDL high density lipoprotein, LDH lactate dehydrogenase, LDL low density lipoprotein, MCV mean corpuscular volume, TG triglycerides, Neu neutrophil.

X-tile analysis illustrated that the optimal cut-off values for 5 years DFS APTT, TT, and APTT/TT were 28.6, 20.6, and 1.4, respectively (**Figures 1a-c**). Based on the cut-off value, the cases were identified into a low APTT/TT groups (<1.4, n=169) and a high (≥1.4, n=95) APTT/TT groups. This suggests that a higher APTT/TT ratio may indicate a poorer prognosis. There was no difference between the optimal cut-off values for ALB, ALP, CHO, FIB, HCT, HDL, LDH, LDL, MCV, TG, and Neu levels as well as DFS (**Figure 2**).

### Comparison of the AUC ability of APTT, TT and APTT/TT

**Figure 3:**
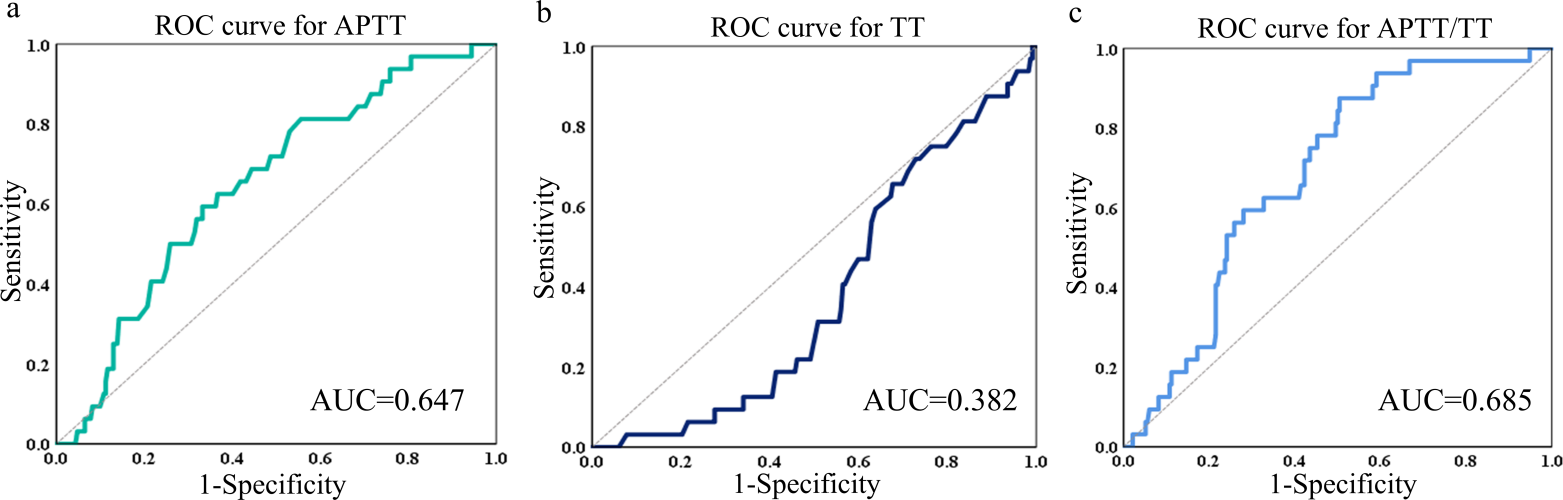
ROC curves of APTT **(a)**, TT **(b)** and APTT/TT **(c)** in predicting the AUC, 95%CI, sensitivity, and specificity. ROC receiver operating characteristic, AUC area under the curve, CI confidence interval.

The Receiver Operating Characteristic (ROC) curve analysis for APTT revealed an Area Under the Curve (AUC) of 0.637, with a 95% confidence interval (CI) ranging from 0.551 to 0.742 (**Figure 3a**). The sensitivity was 59.38% and the specificity was 66.81%. The analysis of the ROC curve for TT suggested an AUC of 0.382 (95%CI: 0.294-0.470). The sensitivity was 87.9% and the specificity was 40.5% (**Figure 3b**).

The ROC curve analysis for APTT/TT demonstrated an AUC of 0.685, with a 95% CI from 0.602 to 0.768. **Figure 3c** shows that the specificity was 49.57% and the sensitivity was 87.50%.

**Figure 4:**
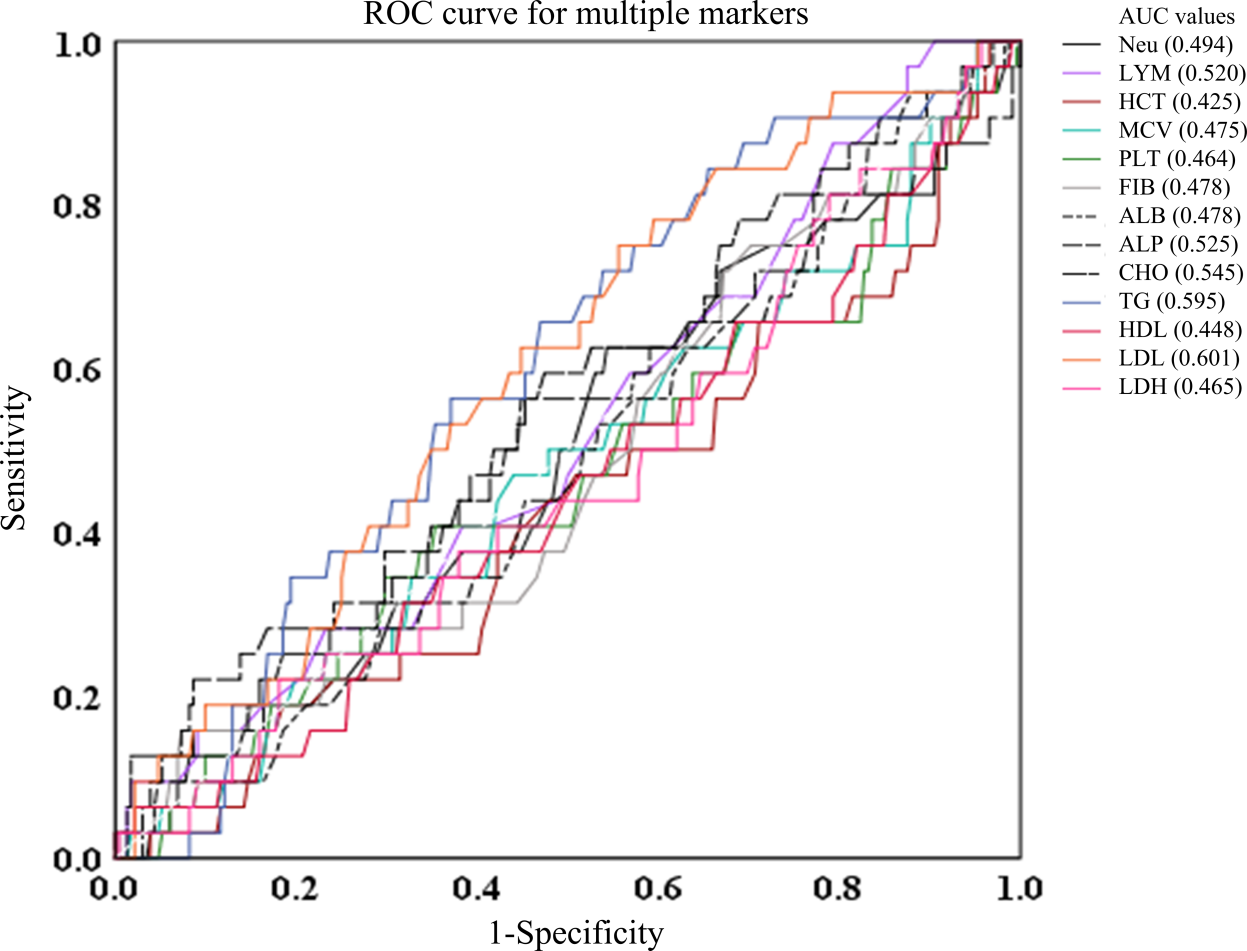
ROC curves of Neu, LYM, HCT, MCV, PLT, FIB, ALB, ALP, CHO, TG, HDL, LDL, and LDH in predicting the AUC, 95%CI, sensitivity, and specificity. ALB albumin, ALP alkaline phosphatase, CHO cholesterol, FIB fibrinogen, HCT hematocrit, HDL high density lipoprotein, LDH lactate dehydrogenase, LDL low density lipoprotein, MCV mean corpuscular volume, TG triglycerides, Neu neutrophil.

Besides, AUCs for Neu, LYM, HCT, MCV, PLT, FIB, ALB, ALP, CHO, TG, HDL, LDL, and LDH were generally low (0.494, 0.52, 0.425, 0.464, 0.478, 0.478, 0.525, 0.545, 0,595, 0.448, 0.601, and 0.465, respectively), suggesting these markers have negligible predictive value for survival events. **Figure 4** is a representation of these findings.

### DFS in the high and low groups of APTT, TT and APTT/TT

**Figure 5:**
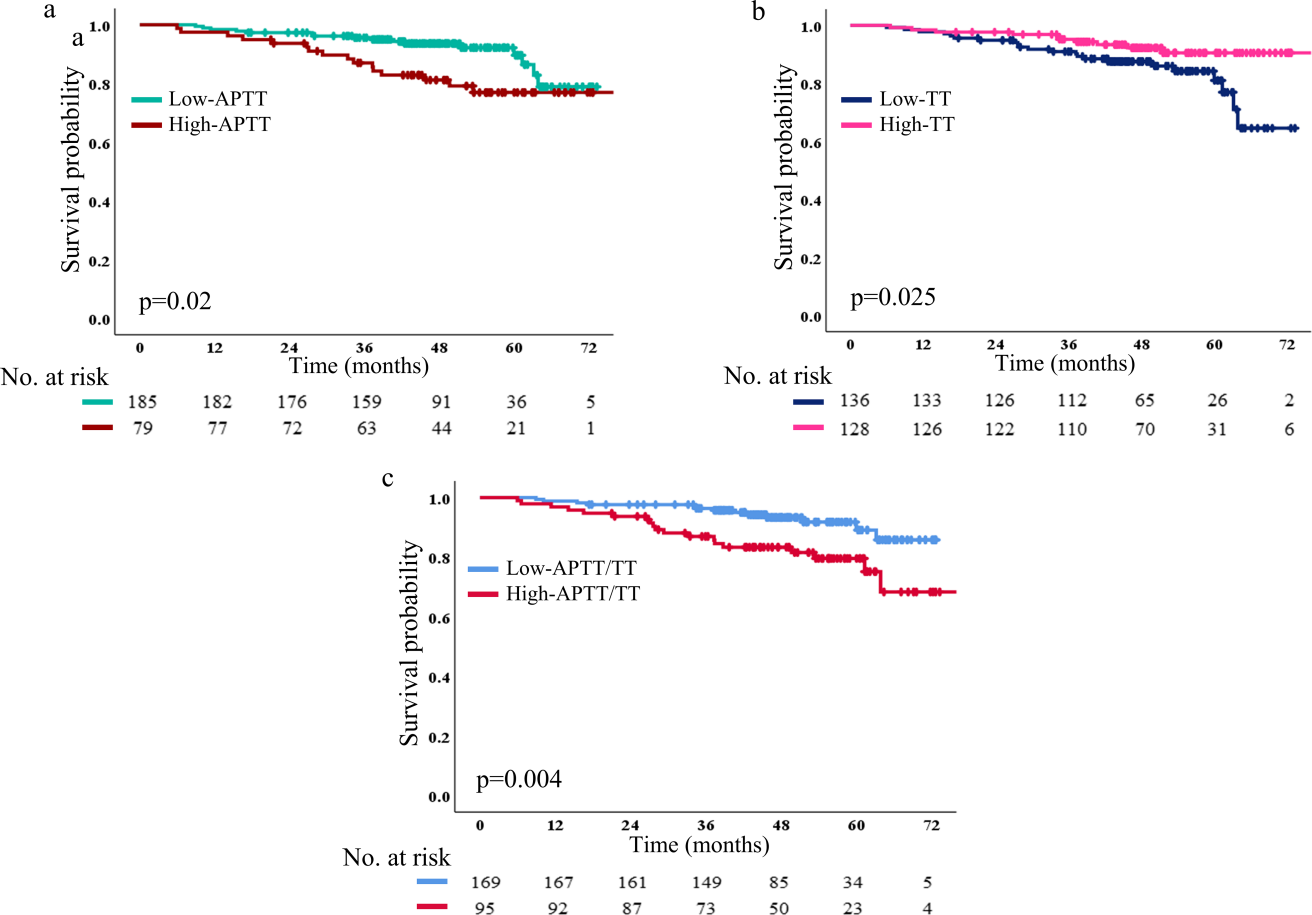
DFS based on low and high APTT **(a)**, TT **(b)**, and APTT/TT **(c)** The number and p-value can be seen clearly. DFS, disease-free survival.

We collected 185 patients (70.1%) in the low APTT (≤28.6) group and 79 patients (29.9%) in the high APTT group (>28.6). The 1-, 2- and 5-year DFS rate in the low APTT group (98.3%, 95.1% and 19.5%, respectively) was significantly longer than that in the high APTT group (97.4%, 91.1% and 26.6%, respectively, p=0.02) (**Figure 5a**).

In **Figure 5b**, it showed that 136 patients (51.5%) in the low TT group and 128 patients (48.5%) in the high TT group. The 1-, 2-, and 5-year DFS rate in the low TT group (97.8%, 92.6% and 19.1%, respectively) was altogether longer than that in high TT group (98.4%, 95.3% and 24.2%, respectively; p=0.025). In addition, 95 patients (36% in the high APTT/TT group) and 169 patients (64% in the low APTT/TT group) were included. The 1-, 2-, and 5-year DFS rate in the low APTT/TT group (98.8%, 95.3% and 20.1%, respectively) was significantly longer than that in high APTT/TT group (96.8%, 91.6% and 24.2%, respectively; p=0.004) (**Figure 5c**). Patients in either the high- or low-level groups failed to reach the median survival time in any of the three survival analyses.

### Multivariate Cox regression analysis for DFS

The effects of various clinical and biochemical variables on DFS in breast cancer patients are examined using univariate and multivariate COX regression models in **Table 2**. Biochemical markers and tumor characteristics are combined in the model.

**Table 2:** Univariate and multivariate Cox regression analysis of DFS.

Factors	Univariable			Multivariable		
	HR	95%CI	p	HR	95%CI	p
Neu	0.950	0.740-1.220	0.688			
APTT	1.104	1.007-1.210	0.034	1.867	0.936-3.726	0.076
TT	0.829	0.714-0.961	0.013	0.434	0.176-1.091	0.076
APTT/TT	7.659	2.008-29.217	0.003	4.057	1.115-14.763	0.034
LYM	1.253	0.679-2.310	0.471			
HCT	0.003	0.000-14.628	0.178			
MCV	0.994	0.924-1.070	0.877			
PLT	0.997	0.991-1.003	0.306			
ALP	1.006	0.990-1.021	0.480			
CHO	1.158	0.801-1.674	0.434			
TG	1.060	0.790-1.424	0.697			
HDL	0.654	0.217-1.975	0.452			
LDL	1.398	0.896-2.183	0.140			
LDH	1.000	0.990-1.009	0.915			
FIB	1.035	0.938-1.142	0.490			
Tumor size	1.646	1.115-2.430	0.012	1.155	0.759-1.758	0.500
Lymph node metastasis	2.520	1.851-3.430	<0.001	2.353	1.708-3.241	<0.001
ER	0.838	0.397-1.771	0.644			
HER-2	0.662	0.297-1.473	0.312			
Ki-67	1.007	0.983-1.031	0.591			

Neu, neutrophil; LYM, lymphocyte; APTT, activated partial thromboplastin time; TT, thrombin time; FIB, Fibrinogen; HCT, hematocrit; MCV, Mean Corpuscular Volume; PLT, platelets; ALP, alkaline phosphatase; CHO, cholestenone; TG, triglyceride; HDL, high density lipoprotein; LDL, low density lipoprotein; LDH, lactic dehydrogenase; FIB, fibrinogen.

Univariable analyses demonstrated that APTT, TT, APTT/TT, tumor size, and LNMs were significantly correlated with DFS (HR:.1.104, 95%CI: 1.008-1.209, p*=*0.034; HR: 0.829, 95%CI: 0.714-0.961, p=0.013; HR: 7.626, 95%CI: 2.006-28.985, p=0.003; HR: 1.646, 95%CI: 1.115-2.430, p=0.012; HR: 2.520, 95%CI: 1.851-3.430, p<0.001), but there were no DFS differences in HCT, MCV, PLT, FIB, ER status, her-2, Ki-67 and other levels (**Table 2**). APTT/TT (HR:4.057, 95%CI: 1.115-14.763, p*=*0.034) and LNMs (HR:2.324, 95%CI: 1.689-3.198, p<0.001) were found to be independent predictors of DFS in the multivariate COX regression model.

### Classification characteristics of APTT, TT, and APTT in LNMs

**Figure 6:**
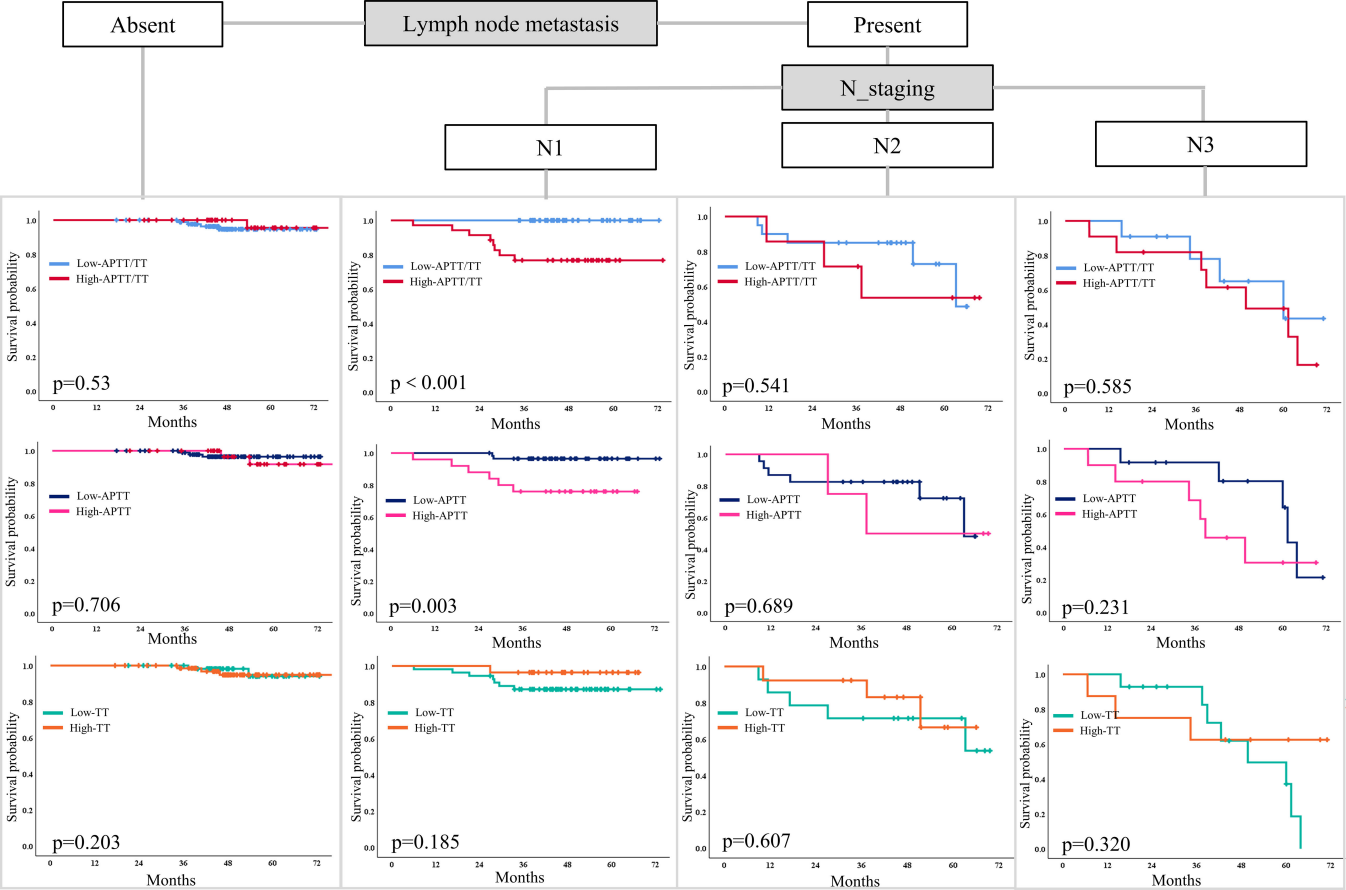
A Kaplan-Meier curve model comparing the hierarchical association of prediction factors associated with DFS among patients with LNMs. DFS disease-free survival, LNM lymph node metastases.

Additionally, patients with LNMs in the high APTT and high APTT/TT groups had shorter survival times (**Figure 6**). Especially in patients with N1 metastasis, these patient in the high APTT (>28.6) and high ATPP/TT (>1.4) had had more limited survival (p=0.001; p=0.003). Thusly, patients with LNMs (N1) with high APTT (>28.6), and high ATPP/TT (>1.4) after first-line treatment had a short DFS. This proposes that the combination of clinicopathological factors and compound coagulation indexes is more helpful for clinical expectation. but N0 and N2-N3 patients did not. In N0-N3, however, there was no difference in survival time between the low and high TT groups.

## Discussion

Our study’s findings emphasize the potential prognostic value of the coagulation index in predicting breast cancer patients’ outcomes after comprehensive treatment. The current understanding of how explicit coagulation index markers can be used to assess patient’s prognosis following treatment is supported and expanded by our research.

Firstly, this affiliation is maintained by broad research showing that abnormal coagulating tests are frequently connected with advanced disease and poorer clinical outcomes in malignant growth patients. Adelborg K. et al. (9) proposed that coagulation factors reflect potential malignancy and may contribute to tumor progression through mechanisms such as promoting metastasis and spread. This is especially obvious from the way that coagulation factors like thrombin act as markers as well as effectively participate in cancer pathology by encouraging angiogenesis and shielding tumor cells from being destroyed by the immune system (10). Giaccherini et al. (11–13) described how thrombin associates with cancer cells to advance cancer development and metastasis. The ability of this connection to serve as a therapeutic goal for thrombin suggests that directing its action might have an impact on the movement of malignant growth and patient survival. Additionally, a growing body of research demonstrates that specific thresholds for clotting markers can classify patients into various prognostic groups and predict patient outcome. The development of an individual treatment plan is aided by this layering. According to study (14), breast cancer patients’ 3-year DFS was influenced by elevated levels of APTT and TT (p < 0.05). Multivariate COX regression analysis showed that APTT and TT were independent prognostic factors affecting 3 years DFS (P < 0.05). However, it is still unclear whether coagulation index can effectively predict prognosis of breast cancer patients after comprehensive treatment. Our study shows that higher APTT/TT ratios are associated with shorter survival in breast cancer patients following comprehensive treatment, similar to findings from a small number of previous studies.

Then, the influence of tumor size and lymph node involvement on survival outcomes has been legitimate in breast cancer research. These factors are critical components of the TNM staging system, which remains a cornerstone for assessing disease severity and prognosis. The study by Q Xu et al. (15) emphaszed that bigger tumor size and extensive lymph node involvement are associated with poorer survival rates, reflecting more aggressive disease states. Essentially, O Menyhárt, et al. (16) elucidated how these physical characteristics of the tumor interact with molecular and biochemical markers to provide a comprehensive prognosis outlook. In our study, univariate analysis showed that APTT, TT, APTT/TT, tumor size, and LNMs were prognostic factors for DFS, while multivariate analysis showed that APTT/TT ratio and LNMs were independent prognostic factors, which was similar to the results of previous studies. However, this study did not discover that tumor size was an independent prognostic factor for survival, which might have been influenced by the patients in this study receiving posterior line therapies.

Furthermore, hormonal and growth factor receptors have a significant impact on the prognosis of breast cancer. The extensive oncology literature, which identifies these receptors as not only diagnostic markers but also key prognostic factors, supports the distinct effects of ER and HER2 on survival (17). ER positivity typically suggests a favorable prognosis due to the availability of targeted therapies like hormone therapy. However, in some aggressive breast cancer subtypes, ER positivity could be linked to resistance to conventional treatments, thereby increasing the risk of unfortunate outcomes. Conversely, HER2 positivity, once considered a poor prognostic indicator, has been transformed into a therapeutic target, significantly improving outcomes for patients with HER2-positive tumors. Studies (18, 19) have shown that explored the protective effect of HER-2 targeted therapy, with lasting benefits for survival. In contrast to the findings of previous studies, the results of this study found that patients with ER-positive and HER-2 amplification were not prognostic factors for DFS. The possible reasons for this result are as follows: 1. The comprehensive treatments in this study were mostly chemotherapy, combined with targeted or endocrine therapy according to ER and HER-2. The premise of this treatment model is aggressive breast cancer. ER positive cannot benefit from chemotherapy early; 2. Since patients with HER-2 amplification receive targeted therapy, negative her-2 does not straightforwardly predict the prognosis of patients.

The study has some limitations. The small number of cases, the short follow-up time, and the limitations of the electronic medical record system are the biggest limitations of this article. In addition, the absence of a healthy control group limits the interpretability of the optimal cut-off values for coagulation markers determined using X-tile analysis. Including baseline data from individuals without breast cancer would enhance the robustness of our findings by offering a comparative reference. However, this was not feasible given the nature of our dataset. Future prospective studies should incorporate control populations for more comprehensive biomarker validation. Despite these difficulties, the depth and breadth of data in retrospective studies provide a solid foundation for the generation of hypotheses that can be tested in prospective studies. Although retrospective studies like ours offer valuable insights by analyzing extensive datasets, they are inherently limited by potential biases like selection bias and information bias. Future prospective studies with larger, multi-center cohorts are warranted to validate and extend our findings. A shift toward incorporating these biological markers into personalized treatment plans appears to be the direction that biomarker research in oncology will take in the foreseeable future.

In conclusion, our research has substantiated the pivotal role of the APTT/TT ratio in forecasting the prognosis of breast cancer patients following comprehensive treatment. The findings underscore the utility of integrating coagulation markers, alongside traditional clinicopathological indices, to enhance prognostic assessments in clinical practice. Notably, increased APTT/TT ratios and more extensive lymph node metastases emerged as independent predictors of diminished disease-free survival, suggesting that these markers could serve as crucial indicators in refining patient management strategies.

## Data Availability

The raw data supporting the conclusions of this article will be made available by the authors, without undue reservation.
